# Skin cancer - what general dental practitioners should look for

**DOI:** 10.1038/s41415-024-7084-6

**Published:** 2024-02-23

**Authors:** Daniel R. Drodge, Konrad Staines, Debbie Shipley

**Affiliations:** 4141518908001 Speciality Registrar, Department of Dermatology, Bristol Royal Infirmary, 1 Marlborough Hill Pl, Bristol, BS2 8HA, UK; 4141518908002 Consultant and Honorary Professor in Oral Medicine, Department of Oral Medicine, Bristol Dental Hospital, Lower Maudlin Street, Bristol, BS1 2LY, UK; Bristol Dental School, 1 Trinity Quay, Avon Street, Bristol, BS2 OPT, UK; 4141518908003 Consultant Dermatologist and Honorary Senior Clinical Lecturer, Department of Dermatology, Bristol Royal Infirmary, 1 Marlborough Hill Pl, Bristol, BS2 8HA, UK

## Abstract

General dental practitioners (GDPs) are well-placed to identify incidental skin lesions when they see patients for routine dental care. Indeed, some patients with an undiagnosed skin malignancy may only see their GDP on a regular basis rather than their general medical practitioner (GMP). GDPs should be able to assess exposed areas of skin, particularly focusing on the head and neck, to identify any lesions of concern and liaise with the patient's GMP where appropriate. We provide an overview focused upon the clinical appearances of isolated benign and malignant lesions, tailored for GDPs.

## Key points

- General dental practitioners are well-placed to notice and act on skin cancers.
- The ABCDE and seven-point checklist are prompts for features of melanoma and can be applied as an aide-mémoire.
- Keratinocyte skin cancers are common and some examples are illustrated in the article.

## Introduction

Skin cancers, comprising basal cell carcinoma (BCC), squamous cell carcinoma (SCC) and melanoma, are the most common of all malignancies.^1^ Many skin cancers arise due to ultraviolet (UV) radiation, with the face being the highest incidence site in one study.^2^ Few health care professionals get as close to the head and neck as general dental practitioners (GDPs), and for some, dental check-ups may be their only regular contact with health professionals.^3^ This presents an opportunity to identify possible skin cancers and refer accordingly, potentially expediting treatment where patients may otherwise delay seeking help until the lesion becomes symptomatic.

This article outlines how to assess and describe skin lesions, then gives an overview of common lesions, under the categories of pigmented and non-pigmented, aiming for familiarity with the common types for a GDP readership, rather than a comprehensive review. We also suggest how to refer lesions from the dental setting in the context of the UK health care setting.

## History of skin lesions

History helps to establish the likelihood that a suspicious skin finding is malignant. Onset and rate of growth are important, although patients may not be able to comment on this, particularly if the lesion is on the back of the head. Symptoms of pain (or tenderness to touch), bleeding and itching should be elicited if not volunteered, as they suggest a growing, invasive or irritated lesion.^4^

Risk factors for skin cancer include personal or family history of skin cancer, history of UV exposure, immunosuppression and the skin phototype (Fitzpatrick type). UV exposure is the main risk factor for skin cancer and is usually inferred from occupational and social history.^5^ Outdoor work (for example, farming, construction) and leisure behaviours (sunbathing, outdoor sports and activities) are relevant, as is having lived in a sunny country. Sunbed use should be asked about. Prior episodes of sunburn, particularly in childhood, are known to increase risk.^6^

Even apparently low-risk individuals with excellent sun protection habits can still develop skin cancers and sometimes history is not available or forthcoming, so an awareness of what benign lesions and skin cancers look like is also important.^7^

## Describing skin lesions

Lesions should be assessed in good light with the full extent of the affected skin visible and palpated for texture, tenderness, consistency and mobility. If feasible, photograph the lesion - the patient may be able to do so on their own mobile device, for later presentation to their general medical practitioner (GMP).

Providing a description is helpful when documenting and making a referral as it provides correlation if photographs are unavailable or the patient is unable to later identify the lesion. Site, size and laterality should be specified. Lesion morphology uses specific terminology: flat; impalpable skin changes (for example, freckles or a bruise) are macules if less than 5 mm or patches if larger than this. Palpable lesions are papules if under 5 mm across, nodules if larger and raised, or plaques if larger but flat-topped.^8^^,^^9^

Further description includes colour and surface characteristics. Pigment, if present, can give rise to brown, black and blue coloration. Note any colour variation within the lesion, for example, multiple shades of brown, loss of pigment and asymmetry. Is there erythema (redness that blanches to touch) and telangiectasia (prominent vessels)? These vascular features are more visible in white skin, sometimes appearing purple or brown in skin of colour. Surface changes can include scale (loose or adherent keratin) and crusting (yellow-brown clot or exudate, indicating underlying erosion or ulceration). Also note any widespread changes beyond the lesion, such as inflammation.^8^

## Pigmented lesions

Pigment derives from melanin, produced by melanocytes and transported to keratinocytes. Increased pigmentation arises physiologically from increased melanin production with stable melanocyte numbers, but change due to melanocyte proliferation is pathological. Physiological causes include facultative pigmentation (tanning) and ephelides (freckles), which are common on sun-exposed sites, especially in children with red hair. These fade in winter, unlike a solar lentigo, which persists, denoting sun damage.^10^

Pigmented lesions are broadly divided into melanocytic or non-melanocytic.^11^ Melanocytic lesions arise from melanocyte overgrowth. Naevi (moles) are benign and are categorised as congenital if present at birth or acquired if manifesting afterwards (usually childhood or young adulthood). The depth of the naevus determines its form - intradermal naevi occur within the dermis; junctional naevi arise at the dermo-epidermal junction. Naevi located deep within the dermis appear blue-black and are termed blue naevi. Facial naevi typically begin as flat and brown in childhood, becoming raised and flesh-coloured in adulthood; it is unlikely that a new melanocytic lesion appearing after the age of 40 is harmless.^12^

Benign naevi are typically single-colour, round, well-demarcated and are similar-looking in the same individual. Naevi that deviate from this form are termed atypical naevi, and on histology, may be found to be dysplastic. They are still classed as benign but have an increased potential to become a malignant melanoma (MM).^13^

MM more often arise *de novo* than develop from existing moles.^14^ The risk of having a melanoma rises steadily from the age of 20 to 60 when the incidence is around 90 per 100,000 population in the UK, increasing thereafter rapidly with over 25% of new cases diagnosed in people aged 75 and over.^14^ MM has the potential to spread to other skin sites, lymph nodes, lung, bone and the central nervous system. Immunotherapy for advanced melanoma has improved survival rates over recent decades, but the optimal treatment remains early detection and surgical excision.^15^

An *in situ* form of melanoma often seen on the face is lentigo maligna. On breaching the dermis, this becomes lentigo maligna melanoma - the risk of it doing so is 5% per year.^16^ As such, lesions suspicious for lentigo maligna warrant referral and consideration of removal. MM can also present in an amelanotic form with no pigment. Their appearance, of a pale or pink plaque or nodule, often with vascular markings, may lead to assessment as possible basal or SCC.^4^

**Fig. 1:**
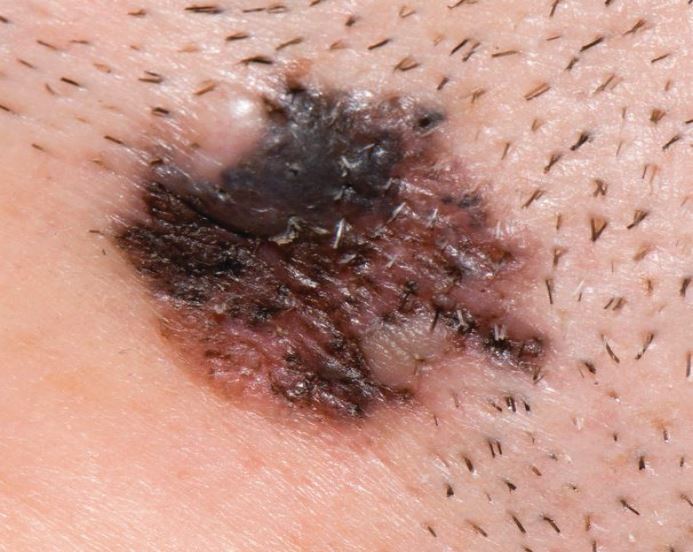
Malignant melanoma

The features of MM are asymmetry, irregularity of border and chaotic distribution of colour, as typified by Figure 1. The ABCDE (asymmetry, border, colour, diameter, evolving) criteria (Table 1) reflect this.^13^ A validated risk-stratification tool is the seven-point weighted screen (see Table 2), which also includes the late features of ulceration and altered sensation. Both serve as an *aide-mémoire* for concerning features when assessing a pigmented lesion. Another useful feature in identifying MM is the ‘ugly duckling' sign - they look different to the patient's other naevi.

**Table 1:** ABCDE criteria and examples of features^13^

Feature	Concerning	Reassuring
Asymmetry	Asymmetry of shape, asymmetric distribution of colour or texture	Round profile, uniformity
Border	Ragged, poorly-defined	Well-demarcated
Colour variegation	More than one colour present	Single colour
Diameter	6 mm or larger at widest point	Smaller
Evolution	Enlarging or changing over course of weeks to months	Stable appearance

**Table 2:** Weighted seven-point checklist, from National Institute for Health and Care Excellence guidance. A score of three or more is stated to warrant referral for suspected skin cancer^25^^,^^26^

Major features (scoring two points each)	Minor features (scoring one point each)
Change in size Irregular shape Irregular colour.	Largest diameter 7 mm or more Inflammation Oozing Change in sensation.

### Seborrhoeic keratosis

**Fig. 2:**
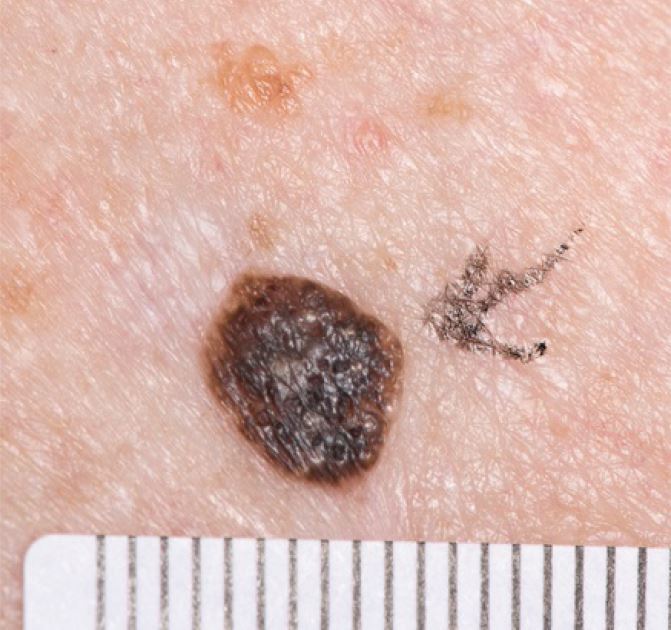
Seborrhoeic keratosis

Seborrhoeic keratosis (SK) is a common benign keratinocyte tumour, illustrated in Figure 2. Around 90% of adults aged over 60 will have at least one SK, which usually start appearing at age 30-40. They can be pigmented, asymmetrical, and if traumatised, can itch, bleed and change appearance due to inflammation. These will thus score on some aspect of ABCDE and raise suspicion of melanoma.^17^ In their typical form, they have a well-demarcated, plaque-like profile (appearing ‘stuck-on'). They are rarely solitary and have similar appearance to other ones on the same individual.^18^ A variant of SK seen in darker skin types is dermatosis papulosa nigra, seen as 1-5 mm multiple brown or dark brown papules on the face, neck and trunk. Onset is in puberty and lesions increase over time. There is a 10-30% prevalence in the Black population.^19^

## Non-pigmented lesions

The majority of skin cancers are non-melanoma and derive instead from keratinocytes. These cells begin in the basal layer of the epidermis, differentiating into their squamous form with keratin-production as they rise towards the skin surface, ending up as flattened, anuclear corneocytes on the outer layer of the epidermis. Dysplastic and malignant keratinocytes can cease keratin production, making them weak and prone to ulcerate, and increase keratin production resulting in scale or even horn-like projections. In less pigmented skin, UV exposure is the causative agent, with sun-exposed sites favoured - photodamage in these areas is evident in the form of wrinkles, solar lentigo and actinic keratosis.

### Red scaly marks

**Fig. 3:**
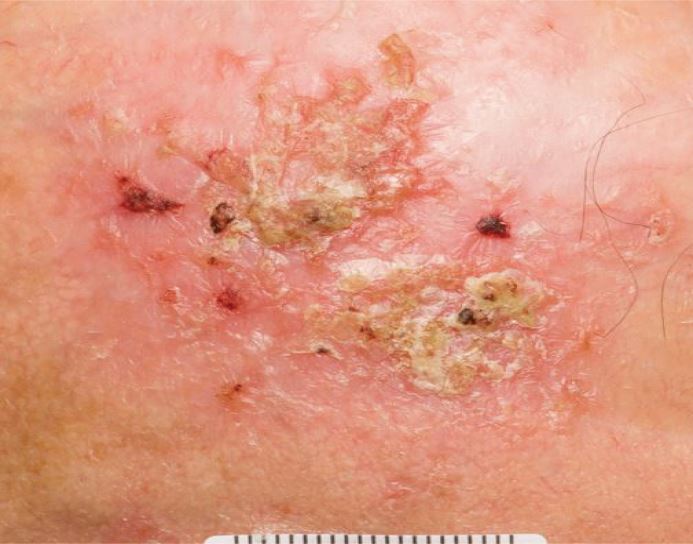
Actinic keratosis of the scalp

Actinic keratosis (AK) is photo-induced squamous cell dysplasia, typically presenting as red, scaly patches (Fig. 3). Keratinous scale is often seen, sometimes resembling horns (hypertrophic AK). These lesions are often found in white, older patients and have a chance of malignant transformation to SCC, so treatment, with cryotherapy, topical chemotherapy or topical anti-inflammatories, is often recommended. Increased thickness, ulceration and pain are suggestive of SCC.

Of particular interest to the GDP is actinic cheilitis (AC) - AK of the lip - which is associated with the same risk factors but a higher risk of malignant progression.^20^ It has been reported that up to 16.9% of AC may progress to SCC. Moreover, the metastasis rate is four times higher for lip SCCs than those elsewhere.^21^ The lower lip is favoured due to increased light exposure. GDPs will have direct vision of the lips and should always be vigilant for atrophy, erythema and scaling, either in a localised or diffuse distribution.

### Pearly, ulcerated or crusted nodules

**Fig. 4:**
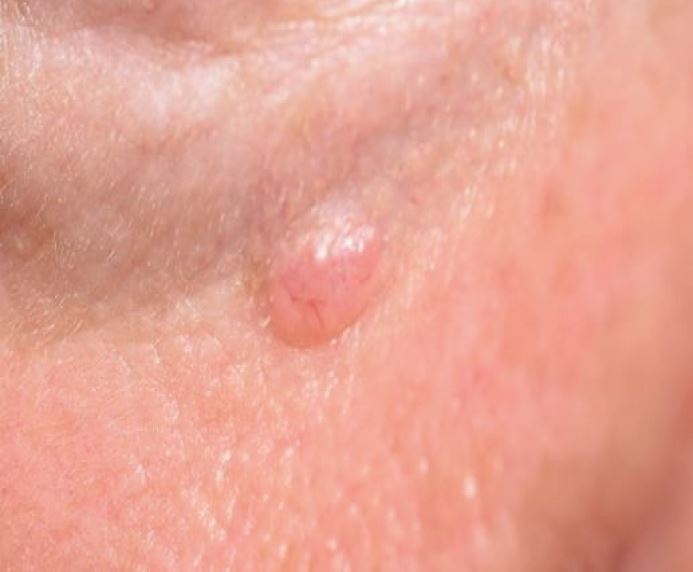
Nodular basal cell carcinoma

**Fig. 5:**
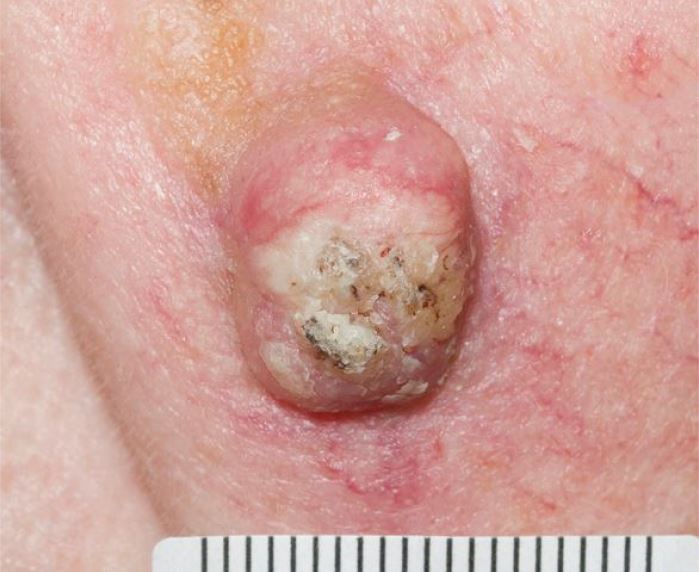
Squamous cell carcinoma - keratotic skin nodule

Current data estimates one in five people will develop a form of keratinocyte cancer - the term encompassing BCC (Fig. 4) and SCC (Fig. 5) - in their lifetime in England. There is a higher risk for men than women.^22^ BCC and SCC are very uncommon in the under 40s, unless there is a genetic predisposition, such as in Gorlin syndrome, or significant immunosuppression, such as in organ transplant recipients. The incidence rises after 40, with the median age of first incidence reported as 71 years for BCC and 79 for cutaneous SCC.

### Basal cell carcinoma

**Fig. 6:**
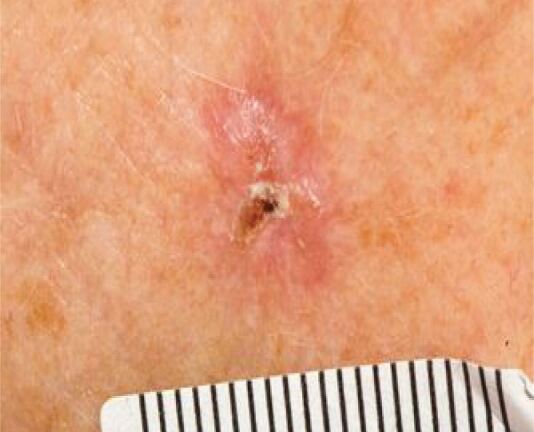
Morphoeic basal cell carcinoma

BCCs arise from the basal keratinocytes. Typically slow-growing, they rarely invade or metastasise. This is the most common of all forms of cancer, with an annual incidence of 283 per 100,000 population in England.^22^ The most common subtype of BCC is nodular - this has a pearly, shiny, rolled edge and arborising telangiectasia (Fig. 4). Ulceration may occur, usually observed as central yellow or red crust, or reported by patients as a recurring, non-healing sore. Other subtypes include superficial, morphoeic (scar-like) (Fig. 6) and infiltrative - these may lack the pearly edge but often feature telangiectasia.^23^ Pigmented BCCs also occur, sometimes presenting as potential melanomas.^24^ The main reason to diagnose and treat these slower-growing, less invasive tumours is to avoid ulceration and disfigurement, as a large BCC can require significant reconstruction after removal.

### Squamous cell carcinoma

Compared to the BCCs, the SCCs are less common (85 per 100,000 population per year) but have more potential for local invasion and metastasis.^22^ They can be diverse in appearance and less stereotypical in appearance than the BCCs. As such, any nodule on the skin that is indurated (a hardened base), or tender to palpation should raise suspicion. SCCs can be keratin-bearing, producing a central scaly surface or even large horn-like projections. They can also ulcerate and thus be mistaken for BCCs, particularly if they are poorly differentiated and lack the ability to produce keratin.

Organ transplant recipients are at raised risk of SCC and skin surveillance is recommended for all such patients. Incidental detection is welcome, particularly if a new lesion arises between check-ups.^5^

An *in situ* form of SCC is Bowen's disease. This has a red, thickened appearance and typically occurs on the shin, but can also be seen on the head and neck.

A notable differential for SCC is the keratoacanthoma, a keratin-bearing fleshy nodule which appears and grows rapidly and will spontaneously regress. Its appearance is of a ‘volcano-like' profile with significant central keratin plug. This physical description also fits a well-differentiated SCC, so such lesions should be treated as such in most cases with prompt referral for dermatological assessment.

## Vascular lesions

**Fig. 7:**
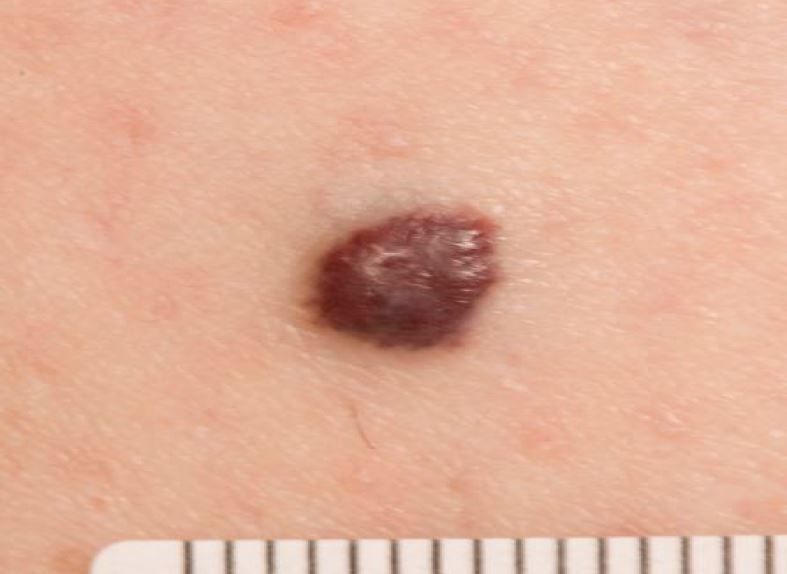
Haemangioma

**Fig. 8:**
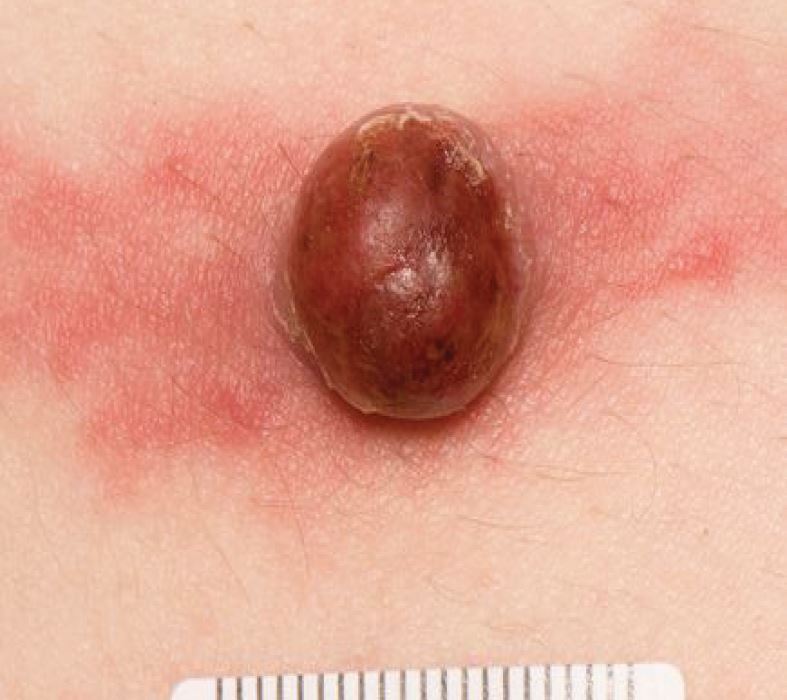
Pyogenic granuloma

Haemangiomas are benign tumours arising from vascular endothelium. A common form, increasing with age, is of small, cherry-red macules or papules which blanch under pressure, known as cherry haemangiomas or Campbell de Morgan spots. We mention haemangiomas here as larger lesions can take on a dark red to black hue and thus appear pigmented (Fig. 7). A variant of note is the lobular capillary haemangioma, or pyogenic granuloma (Fig. 8). This is a nodular vascular tumour, prone to bleeding with minimal provocation, often with a pedunculated form. They can be seen at sites of trauma on the head and neck and often arise in young people during pregnancy. Their rapid growth and recurrent, non-healing bleeding leads to suspicion of malignancy.

## Acting on suspected skin cancer

GDPs discovering lesions suspicious for cancer should advise GMP assessment. The GMP will then decide whether onward referral is warranted, using for example the two-week-wait skin cancer referral pathway. The NHS produces guidance to support this for pigmented lesions, using the weighted seven-point checklist (Table 1).^25^^,^^26^ For non-melanoma skin cancers, all suspected SCCs should be referred, but BCCs are generally referred on a routine basis, reflecting their indolent growth rate and low risk of invasion. Exception is made for a facial lesions or large lesions.

The GDP, in correspondence with the GMP, should aim to include the history of the lesion and clinical appearance, preferably supplemented with clinical photographs. They should also establish follow-up with the patient concerned as a safety net in case of delays in assessment. The patient should be kept informed throughout these discussions, of what has been asked and what to expect.

## Conclusion

Skin cancer is common, arises often on the head and neck and GDPs are well-placed to identify them. Patient history, including risk factors along with physical findings, can provide the basis for suspicion of BCC, SCC or MM. Pigmented lesions raise suspicion of melanoma, but also include benign entities, such as naevi and seborrhoeic keratoses. Non-pigmented lesions may be SCC or BCC but may be actinic keratosis. Vascular lesions tend to appear red to purple but are generally benign. We advocate referral - or advice to self-refer - unless the lesion is confidently identified as benign, but inclusion of clinical detail in the referral can help colleagues in general medical practice and hospital specialties, who will always be grateful for the chance to intervene early.

## References

[1] Urban K, Mehrmal S, Uppal P, Giesey R L, Delost G R. The global burden of skin cancer: A longitudinal analysis from the Global Burden of Disease Study, 1990-2017. *JAAD Int *2021; **2:** 98-108. 10.1016/j.jdin.2020.10.013 PMC8362234 34409358

[2] Ciążyńska M, Kamińska-Winciorek G, Lange D* et al.* The incidence and clinical analysis of non-melanoma skin cancer. *Sci Rep* 2021; **11:** 4337. 10.1038/s41598-021-83502-8 PMC7900109 33619293

[3] Kutcher M J, Rubenstein D. Fifteen inches from cancer: early recognition of facial lesions by the dentist. *Compend Contin Educ Dent* 2004; **25:** 939-942. 15645909

[4] Griffiths C E, Bleiker T O, Creamer D, Ingram J R, Simpson R C. *Rook's Dermatology Handbook*. 1st ed. New Jersey: Wiley Blackwell, 2022.

[5] Stratigos A J, Garbe C, Dessinioti C* et al.* European interdisciplinary guideline on invasive squamous cell carcinoma of the skin: Part 1. epidemiology, diagnostics and prevention. *Eur J Cancer* 2020; **128:** 60-82. 10.1016/j.ejca.2020.01.007 32113941

[6] Leiter U, Keim U, Garbe C. Epidemiology of Skin Cancer: Update 2019. *Adv Exp Med Biol* 2020; **1268:** 123-139. 10.1007/978-3-030-46227-7_6 32918216

[7] Calzavara-Pinton P, Ortel B, Venturini M. Non-melanoma skin cancer, sun exposure and sun protection. *G Ital Dermatol Venereol* 2015; **150:** 369-378. 26186380

[8] James W D, Elston D M, Treat J R, Rosenbach M A*. Andrews' Diseases of the Skin. *Edinburgh: Elsevier, 2020.

[9] Bolognia J, Schaffer J V, Cerroni L. *Dermatology*. Philadelphia: Elsevier, 2018.

[10] Lin J Y, Fisher D E. Melanocyte biology and skin pigmentation. *Nature* 2007; **445:** 843-850. 10.1038/nature05660 17314970

[11] Shain A H, Bastian B C. From melanocytes to melanomas. *Nat Rev Cancer* 2016; **16:** 345-358. 10.1038/nrc.2016.37 27125352

[12] Farabi B, Akay B N, Goldust M, Wollina U, Atak M F, Rao B. Congenital melanocytic naevi: An up-to-date overview. *Australas J Dermatol* 2021; **62:** 178-191. 10.1111/ajd.13535 33591589

[13] Abbasi N R, Shaw H M, Rigel D S* et al.* Early diagnosis of cutaneous melanoma: revisiting the ABCD criteria. *JAMA *2004; **292:** 2771-2776. 10.1001/jama.292.22.2771 15585738

[14] Schadendorf D, van Akkooi A C, Berking C* et al.* Melanoma. *Lancet* 2018; **392:** 971-984. 10.1016/S0140-6736(18)31559-9 30238891

[15] Garbe C, Amaral T, Peris K* et al.* European consensus-based interdisciplinary guideline for melanoma. Part 2: Treatment - Update 2022. *Eur J Cancer* 2022; **170:** 256-284. 10.1016/j.ejca.2022.04.018 35623961

[16] Garbe C, Amaral T, Peris K* et al.* European consensus-based interdisciplinary guideline for melanoma. Part 1: Diagnostics: Update 2022. *Eur J Cancer* 2022; **170:** 236-255. 10.1016/j.ejca.2022.03.008 35570085

[17] Coulson I H, Benton E C, Ogden S. Diagnosis of Skin Disease. *In* Griffiths C E, Barker J, Bleiker T O, Chalmers R, Creamer D (eds) *Rook's Textbook of Dermatology. *9th ed.pp 1-28. New Jersey: Wiley, 2016.

[18] Farrant P, Mowbray M, Sinclair R D. Dermatoses of the Scalp. *In* Griffiths C E, Barker J, Bleiker T O, Chalmers R, Creamer D (eds) *Rook's Textbook of Dermatology. *9th ed pp 1-17. New Jersey: Wiley, 2016.

[19] Li B S, Maibach H I. *Ethnic Skin and Hair and Other Cultural Considerations*. Cham: Springer Cham, 2021.

[20] Trager M H, Farmer K, Ulrich C* et al.* Actinic cheilitis: a systematic review of treatment options. *J Eur Acad Dermatol Venereol* 2021; **35:** 815-823. 10.1111/jdv.16995 33251620

[21] Markopoulos A, Albanidou-Farmaki E, Kayavis I. Actinic cheilitis: clinical and pathologic characteristics in 65 cases. *Oral Dis* 2004; **10:** 212-216. 10.1111/j.1601-0825.2004.01004.x 15196142

[22] Van Bodegraven B, Vernon S, Eversfield C* et al.* ‘Get Data Out' Skin: national cancer registry incidence and survival rates for all registered skin tumour groups for 2013-2019 in England. *Br J Dermatol* 2023; **188:** 777-784. 10.1093/bjd/ljad033 36814132

[23] Smoller B, Bagherani N. *Atlas of Dermatology, Dermatopathology and Venereology.* Cham: Springer Cham, 2022.

[24] Peris K, Fargnoli M C, Garbe C* et al.* Diagnosis and treatment of basal cell carcinoma: European consensus-based interdisciplinary guidelines. *Eur J Cancer* 2019; **118:** 10-34. 10.1016/j.ejca.2019.06.003 31288208

[25] Walter F M, Prevost A T, Vasconcelos J* et al.* Using the 7-point checklist as a diagnostic aid for pigmented skin lesions in general practice: a diagnostic validation study. *Br J Gen Pract* 2013; **63:** 345-353. 10.3399/bjgp13X667213 PMC3635581 23643233

[26] National Institute for Health and Care Excellence. Suspected cancer: recognition and referral. 2015. Available at https://www.nice.org.uk/guidance/ng12/chapter/Recommendations-organised-by-site-of-cancer#skin-cancers (accessed January 2024). 26180880

